# Antioxidant Activities and Polyphenolic Contents of Three Selected *Micromeria* Species from Croatia

**DOI:** 10.3390/molecules16021454

**Published:** 2011-02-10

**Authors:** Sanda Vladimir-Knežević, Biljana Blažeković, Maja Bival Štefan, Antun Alegro, Tamás Kőszegi, József Petrik

**Affiliations:** 1 Department of Pharmacognosy, Faculty of Pharmacy and Biochemistry, University of Zagreb, Marulićev trg 20, HR-10000 Zagreb, Croatia; 2 Department of Botany and Botanical Garden, Faculty of Science, University of Zagreb, Marulićev trg 20, HR-10000 Zagreb, Croatia; 3 Institute of Laboratory Medicine, Medical School, University of Pécs, Ifjúság útja13, H-7624 Pécs, Hungary; 4 Department of Medical Biochemistry and Haematology, Faculty of Pharmacy and Biochemistry, University of Zagreb, Domagojeva 2, HR-10000 Zagreb, Croatia

**Keywords:** *Micromeria croatica*, *Micromeria juliana*, *Micromeria thymifolia*, antioxidant activity, polyphenols, rosmarinic acid

## Abstract

Antioxidant activities of three selected *Micromeria* species growing in Croatia (*M. croatica*, *M. juliana* and *M. thymifolia*) were evaluated using five different antioxidant assays, in comparison with plant polyphenolic constituents and reference antioxidants. All studied ethanolic extracts exhibited considerable activity to scavenge DPPH and hydroxyl free radicals, reducing power, iron chelating ability and total antioxidant capacity in the order: *M. croatica* > *M. juliana* > *M. thymifolia*. Total polyphenol (9.69–13.66%), phenolic acid (5.26–6.84%), flavonoid (0.01–0.09%) and tannin (3.07–6.48%) contents in dried plant samples were determined spectrophotometrically. A strong positive correlation between antioxidant activities and contents of phenolic acids and tannins was found, indicating their responsibility for effectiveness of tested plants. Our findings established *Micromeria* species as a rich source of antioxidant polyphenols, especially the endemic *M. croatica*.

## 1. Introduction

Natural antioxidants have been studied extensively for decades in order to find compounds protecting against a number of diseases related to oxidative stress and free radical-induced damage. Oxidative stress is associated with pathogenetic mechanisms of many diseases including atherosclerosis, neurodegenerative diseases, cancer, diabetes and inflammatory diseases, as well as aging processes. It is defined as an imbalance between production of free radicals and reactive metabolites, so-called oxidants, and it also includes their elimination by protective mechanisms, referred to as antioxidative systems. This imbalance leads to damage of important biomolecules and organs with potential impact on the whole organism. Antioxidants can delay, inhibit or prevent the oxidation of oxidizable materials by scavenging free radicals and diminishing oxidative stress [1,2]. 

The plant kingdom offers a wide range of compounds exhibiting antioxidant activities. Polyphenols have been considered as excellent natural antioxidants. They are widely distributed and can be considered as the most abundant plant secondary metabolites with highly diversified structures [3,4]. Lamiaceae family includes numerous popular and less known herbs with pronounced therapeutic properties. This large botanical family is taxonomically divided into several subfamilies, one of the largest being the Nepetoidae which comprises species that are the most important as sources of antioxidants [5]. The genus *Micromeria* Bentham belongs also to this subfamily. It is represented by 52 species which are distributed from the Macaronesian and Mediterranean region to South Africa, India and China [6]. They are traditionally used against heart disorders, headache, colds, wounds and skin infections, as well as for insecticidal, herbicidal and culinary purposes [7,8]. 

The important representatives of genus *Micromeria* in Croatian flora are *M. croatica* (Pers.) Schott, *M. thymifolia* (Scop.) Fritsch and *M. juliana* (L.) Bentham ex Reichenb. The first two are endemic species in Croatia and in some neighbouring countries, while *M. juliana* is more widely distributed in Mediterranean region [9]. The previous phytochemical investigations of these *Micromeria* species revealed the presence of essential oil, flavonoids and triterpenes [10,11,12,13,14,15,16,17,18]. Most of the studies on these plants were carried out for investigation of volatile compounds and their antimicrobial activities [8,19,20,21,22,23]. To the best of our knowledge, there are no literature data concerning antioxidant properties of *M. croatica* and *M. thymifolia*, while only one study covering antioxidant activity of *M. juliana* was published. Öztürk *et al.* tested various extracts of *M. juliana* for their antioxidant activity by different assays. Light petroleum extract exhibited the strongest lipid peroxidation inhibition effect and the research was mainly focused on the elucidation of its chemical composition and anticholinesterase activity too [24]. 

In search of finding new resources and potent antioxidant, the present study aimed to evaluate antioxidant potential of ethanolic extracts of three selected *Micromeria* species and to measure the impact of polyphenolic constituents on their effectiveness. 

## 2. Results and Discussion

### 2.1. Phytochemical analysis of polyphenolic compounds

**Figure 1:**
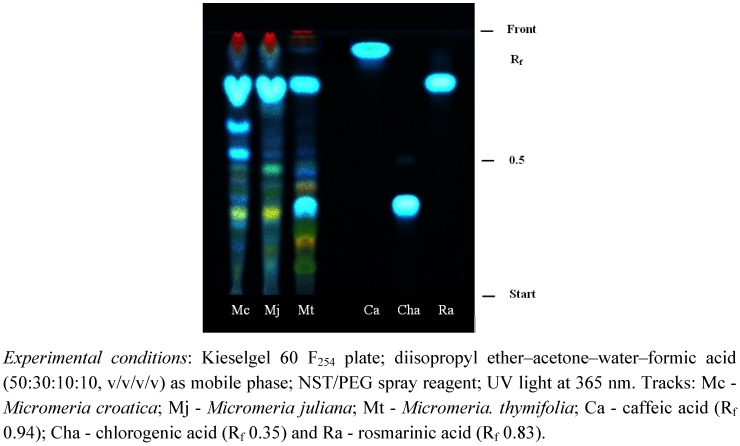
TLC chromatogram of phenolic acids in ethanolic extracts of the three selected *Micromeria* species.

*Micromeria* species were investigated regarding their polyphenolic content and composition. The presence of flavonoids, phenolic acids and tannins in ethanolic plant extracts was detected by thin layer chromatography (TLC). The flavonoids proved to be derivatives of acacetin, apigenin and luteolin being in accordance with the literature data [15,16]. Two phenolic acids were identified as rosmarinic and chlorogenic acid, respectively, verified by their intense blue fluorescent bands as shown in Figure 1 (R_f_ values 0.94 and 0.35, respectively). TLC analysis revealed rosmarinic acid as the predominant compound in *Micromeria* ethanolic extracts. 

The contents of total polyphenols and individual polyphenolic subclasses were determined in aerial plant parts by different colorimetric methods, and the results are shown in Table 1. As can be seen, amounts of total polyphenols in all plant samples were considerable, ranging between 9.69% and 13.06%. The most abundant compounds were phenolic acids (5.26–6.84%), followed by tannins (3.07–6.08%), contrary to low percentage contribution of flavonoids (0.01–0.09%). *M. croatica* contained the highest amounts of phenolic acids and tannins (p < 0.001), whereas their lowest levels were found in *M. thymifolia*, which contained significantly (p < 0.001) greater quantity of flavonoids than other studied plants. These findings were consistent with the results obtained by TLC analysis, indicating the greatest contribution of phenolic acids to the total polyphenolic contents in *Micromeria* species. 

**Table 1:** Contents of phenolic acids, flavonoids, tannins and total polyphenols in three selected *Micromeria* species.

Plant species	Contents (%)			
	Phenolic acids	Flavonoids	Tannins	Total polyphenols
*M. croatica*	6.84 ± 0.06	0.01 ± 0.002	6.08 ± 0.06	13.06 ± 0.08
*M. juliana*	5.42 ± 0.06	0.04 ± 0.003	5.14 ± 0.20	10.75 ± 0.58
*M. thymifolia*	5.26 ± 0.01	0.09 ± 0.020	3.07 ± 0.35	9.69 ± 0.58

Each value is the mean ± SD of three independent measurements.

### 2.2. Antioxidant activities of Micromeria ethanolic extracts

Polyphenolic compounds such as flavonoids, phenolic acids and tannins are considered to be the major contributors to the antioxidant activity of medicinal plants, fruits and vegetables. The antioxidant activities of polyphenols were attributed to their redox properties, which allow them to act as reducing agents, hydrogen donators and singlet oxygen quenchers, as well as their metal chelating abilities [3,25]. Therefore, in the present study five different assays were employed in order to determine and compare the antioxidant properties of selected *Micromeria* species, as well as to elucidate their mode of action. 

The DPPH assay has been widely used to evaluate the free radical scavenging effectiveness of various antioxidant substances. Nitrogen centered radicals such as DPPH^•^ react with phenols via two different mechanisms: direct abstraction of phenol H-atoms and electron transfer processes. The contribution of one or the other pathway depends on the nature of solvent and/or the redox potentials of the species involved. DPPH*^•^* is a stable free radical compound with a characteristic absorption at a wavelength of 517 nm. Antioxidants upon interaction with DPPH^•^ either transfer an electron or hydrogen atom to DPPH^•^, thus neutralizing its free radical character. The colour of the reaction mixture changes from purple to yellow with a decrease of the 517 nm absorbance. The degree of discolouration indicates the scavenging potential of the antioxidants [26,27].

**Figure 2:**
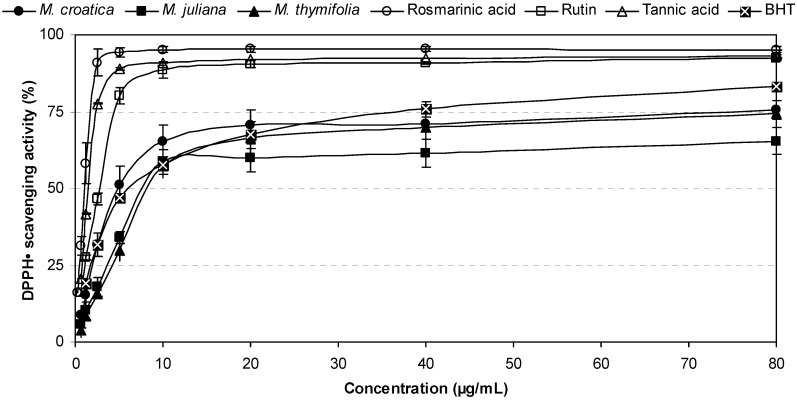
DPPH free radical scavenging effects of *Micromeria* ethanolic extracts in comparison with polyphenolic compounds and a reference antioxidant.

Figure 2 shows the percent inhibition of DPPH^• ^with tested *Micromeria* ethanolic extracts and pure antioxidant compounds at different concentrations (0.30–80 μg/mL). The activities of plant extracts were 16–30%, 30–52% and 57–62% at 2.50 μg/mL, 5 μg/mL and 10 μg/mL, respectively. Rosmarinic acid and rutin showed the highest radical scavenging effectiveness. Activity of rosmarinic acid was between 16% and 58% already at 0.30–1.25 μg/mL. Abilities of the tested samples to scavenge DPPH^•^ were assessed on the basis of their IC_50_ values which were inversely related to their antioxidant capacities, as they express the amount of the antioxidant needed to decrease the radical concentration by 50%. The IC_50_ values obtained in this study are listed in Table 2. The scavenging effects of the studied samples on the DPPH radical decrease in order of rosmarinic acid > rutin > *M. croatica* > BHT > *M. juliana* > *M. thymifolia*. Among the tested plant samples, ethanolic extract of *M. croatica* exhibited the most effective radical scavenging activity (IC_50_ = 4.67 μg/mL) which was also significantly stronger (p < 0.05) than that of butyated hydroxytoluene (BHT; IC_50_ = 6.45 μg/mL). This can be attributed mainly to higher phenolic amount of *M. croatica*, especially phenolic acids, than of other plant samples. Our results also accentuated rosmarinic acid as a powerful free radical scavenger (IC_50_ = 1.06 μg/mL). 

The hydroxyl radical is an extremely reactive oxygen species, capable of modifying several biologically important molecules in the living cells. This radical is able to cause DNA damages leading to carcinogenesis, mutagenesis and cytotoxicity. Moreover, hydroxyl radicals are capable of initiating the process of lipid peroxidation by abstracting hydrogen atoms from unsaturated fatty acids. They can also abstract hydrogen atoms from other biological molecules, including thiols, leading to the formation of sulphur radicals being able to combine with oxygen to generate oxysulphur radicals, some of which damage biological molecules [28]. Due to their high reactivity, in this study the hydroxyl radical scavenging activities of *Micromeria* extracts were determined using deoxyribose assay. 2-Deoxy-D-ribose is oxidized when exposed to hydroxyl radicals generated by Fenton-type reaction. In this case hydroxyl radicals produced by decomposition of H_2_O_2_ by high redox potential of EDTA–Fe^2+^ complex attack deoxyribose and cause its fragmentation. The oxidative degradation can be detected by heating the products with 2-thiobarbituric acid (TBA) under acid conditions to develop a pink chromogen (thiobarbituric acid reactive species, TBARS) with a maximum absorbance wavelength of 532 nm. Added hydroxyl radical scavengers compete with deoxyribose for hydroxyl radicals and diminish chromogen formation [29]. 

**Figure 3:**
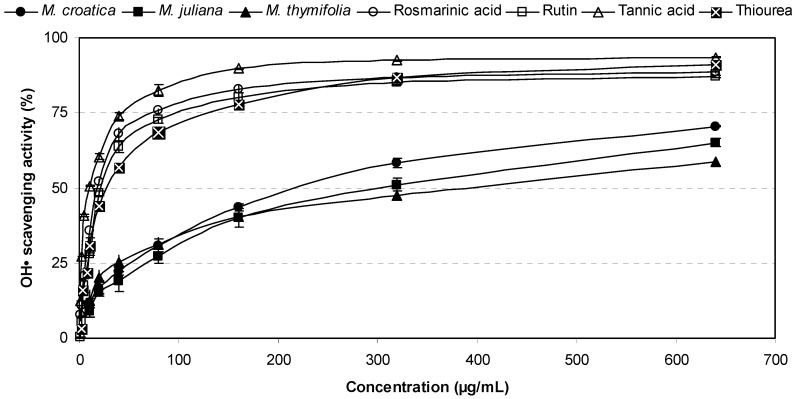
OH free radical scavenging effects of *Micromeria* ethanolic extracts in comparison with polyphenolic compounds and a reference antioxidant.

The radical scavenging abilities of *Micromeria* ethanolic extracts, polyphenolic compounds and a reference antioxidant are presented in Figure 3. All tested extracts exerted inhibitory activities on the hydroxyl radical formation in a concentration dependant manner. They scavenged OH^•^ by 27–33%, 41–44% and 48–58% at 80 μg/mL, 160 μg/mL and 320 μg/mL, respectively. These values were about twofold lower than that of the same dose of thiourea, which is a well-known antioxidant. Among the studied plants, *M. croatica* showed the highest activity, with an IC_50 _value of 249.65 μg/mL (Table 2) and these results were in accordance with findings obtained by the DPPH assay. Moreover, the scavenging abilities on hydroxyl radicals were in the same descending order: *M. croatica* > *M. juliana* > *M. thymifolia*. Tannic acid, rosmarinic acid and rutin with IC_50 _values 9.63 μg/mL, 18.61 μg/mL and 22.25 μg/mL, respectively, showed significantly stronger effects (p < 0.001) than thiourea (IC_50 _= 31.14 μg/mL). This assay demonstrated that *Micromeria* extracts could control the quantity of aggressive OH^• ^owing to their polyphenolic constituents. 

Many studies have suggested that the electron donating capacity, reflecting the reducing power of bioactive compounds, is associated with antioxidant activity. Reducing power is generally associated with the presence of reductones which exert antioxidant action by breaking the free radical chain. Besides, reductones can reduce the oxidized intermediates of lipid peroxidation processes, so that they can act as primary and secondary antioxidants. The reducing power assay measures the electron-donating ability of antioxidants using the potassium ferricyanide reduction method. Antioxidants cause the reduction of the Fe^3+^/ferricyanide complex to the ferrous form and activity is measured as the increase in the absorbance at 700 nm. In this assay, the yellow colour of the test solution changes to various shades of green and blue depending on the reducing power of antioxidant samples [30,31,32]. 

**Figure 4:**
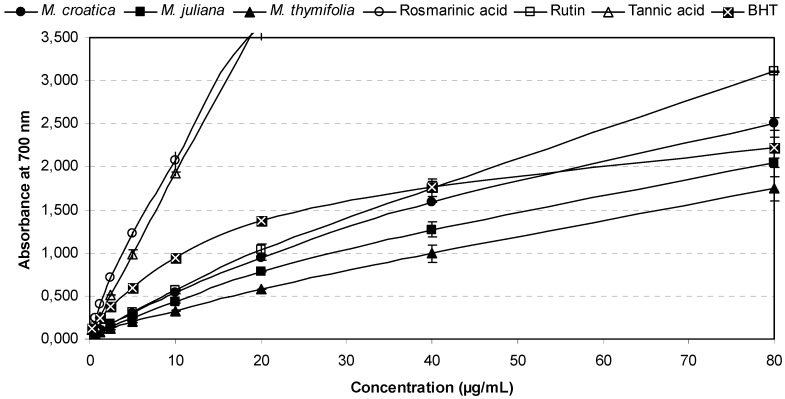
Reducing power of *Micromeria* ethanolic extracts in comparison with polyphenolic compounds and a reference antioxidant.

Figure 4 shows the plot of reducing power of *Micromeria* ethanolic extracts in comparison with polyphenolic compounds and BHT as a reference antioxidant. At tested concentrations (0.63–80 μg/mL) all samples possessed the ability to reduce iron(III) ions. The reducing power of the plant extracts increased with concentrations in a strongly linear manner (R^2^ = 0.9785–0.9946). In this assay, *M. croatica* exhibited once again the most powerful effect (IC_50 _= 9.64 μg/mL) in comparison with *M. juliana* (IC_50 _= 12.38 μg/mL) and *M. thymifolia* (IC_50 _= 17.46 μg/mL) (Table 2). Reducing power of *M. croatica* extract at concentrations up to 40 μg/mL was lower than that of BHT, but at 80 μg/mL slightly exceeded activity of the reference antioxidant. Rosmarinic acid and tannic acid with IC_50 _values of 1.64 μg/mL and 2.47 μg/mL, respectively, demonstrated the strongest reducing properties, even significantly stronger (p < 0.001) than BHT (IC_50 _= 4.07 μg/mL). Our results are in agreement with previous research on rosmarinic and tannic acids [33,34] and suggest a huge influence of rosmarinic acid on the reductive capability of tested *Micromeria* extracts. 

The antioxidant activities of phenolic compounds are also attributed to their ability to chelate transition metal ions, such as those of iron and copper, which have been proposed as the catalysts for the initial formation of reactive oxygen species. Chelating agents may stabilize pro-oxidative metal ions in living systems by complexing them [3,35]. Iron(II) ion is known as a potent inducer of lipid peroxidation. It accelerates lipid oxidation by breaking down hydrogen and lipid peroxides to reactive free radicals via the Fenton type reaction. Ferrozine can quantitatively form complexes with Fe^2+^. In the presence of chelating agents, the complex formation is disrupted resulting in a decrease in the red colour of the complex. Measurement of the colour intensity reduction at the 562 nm wavelength allows estimation of the metal chelating activity of the coexisting chelator [30,31]. In this respect, *Micromeria* ethanolic extracts were assessed for their ability to compete with ferrozine for Fe^2+^ in the solution. 

**Figure 5:**
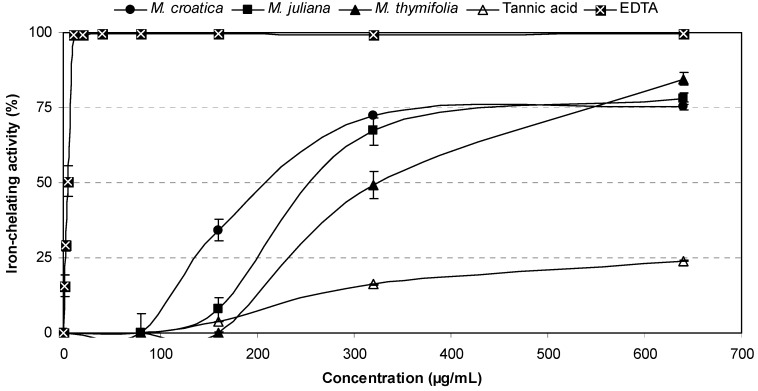
Iron(II) ions chelating activities of *Micromeria* ethanolic extracts in comparison with tannic acid and a reference chelator.

All extracts demonstrated an ability to chelate Fe^2+^ at concentrations higher than 80 μg/mL (Figure 5). *M. croatica* extract at concentrations up to 320 μg/mL appeared to be a considerably better chelator (34–72%) than the other tested plant extracts, but at about 560 μg/mL all samples showed equivalent chelating activities of approximately 75%. Table 2 reveals no distinctive difference between the IC_50 _values of *M. croatica* (227.47 μg/mL) and *M. juliana* (254.33 μg/mL), but these values were significantly lower (p < 0.001) than that of *M. thymifolia* (336.33 μg/mL). However, the chelating abilities of *Micromeria* extracts were much lower than EDTA which is one of the most powerful metal chelator ever known (IC_50 _= 4.78 μg/mL). Nevertheless, in this assay tannic acid was found to be a very weak chelator of iron(II) ions, while rosmarinic acid and rutin at tested concentrations showed no activity at all (Table 2). These findings indicate that chelating abilities of *Micromeria* ethanolic extracts do not depend on the presence of rosmarinic acid, and it is quite possible that their effectiveness is attributed to the other phenolic compounds contained. 

Total antioxidant capacities of *Micromeria* ethanolic extracts in comparison with polyphenolic compounds and a reference antioxidant were evaluated by phosphomolybdenum method. This assay is based on the reduction of Mo(VI) to Mo(V) by the antioxidant compounds and the subsequent formation of a green phosphate/Mo(V) complex at acidic pH with a maximal absorption at 695 nm [36].

**Figure 6:**
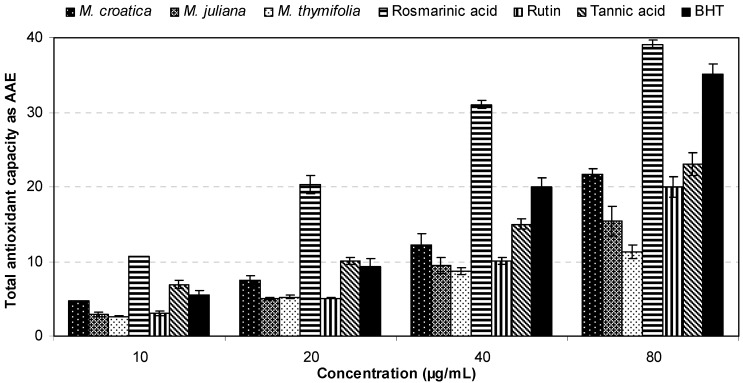
Total antioxidant capacities of *Micromeria* ethanolic extracts in comparison with polyphenolic compounds and a reference antioxidant.

The obtained results, expressed as ascorbic acid equivalents (AAE), are presented in Figure 6 and Table 2. All investigated samples were active in a concentration dependent manner and their potency decreased in the following order: rosmarinic acid > BHT > tannic acid > *M. croatica* > rutin > *M. juliana* > *M. thymifolia*. Among the tested *Micromeria* species, the highest total antioxidant capacity was seen for *M. croatica* with a value of 470.03 mg AAE per gram of dry extract.

The total antioxidant capacity of rosmarinic acid was found to be much higher (1072.40 mg AAE/g) when compared to BHT (694.07 mg AAE/g), emphasizing its huge antioxidant potential. According to these results, rosmarinic acid was confirmed as the most important contributor to the overall antioxidant effectiveness of *Micromeria* ethanolic extracts, as previously suggested by the most of antioxidant assays performed in this study.

**Table 2:** Comparative overview of IC_50_ values as well as total antioxidant capacities of *Micromeria* ethanolic extracts, polyphenolic compounds and reference antioxidants.

Samples	IC_50_^*^ (µg/mL)				Total antioxidant capacity (mg AAE/g)^**^
	DPPH^·^scavenging activity	OH^•^ scavenging activity	Reducing power	Iron chelating activity	
*M. croatica*	4.67 ± 0.57	249.65 ± 1.64	9.64 ± 0.40	227.47 ± 13.06	470.03 ± 20.58
*M. juliana*	7.95 ± 0.20	324.03 ± 12.16	12.38 ± 0.20	254.33 ± 16.17	284.50 ± 10.12
*M. thymifolia*	8.33 ± 0.72	390.98 ± 32.05	17.46 ± 1.41	336.33 ± 21.83	265.76 ± 4.82
Rosmarinic acid	1.06 ± 0.13	18.61 ± 0.35	1.64 ± 0.00	nd	1072.40 ± 11.20
Rutin	2.82 ± 0.16	22.25 ± 1.03	8.69 ± 0.30	nd	304.09 ± 27.71
Tannic acid	1.54 ± 0.09	9.63 ± 1.06	2.47 ± 0.16	nd	694.07 ± 31.59
BHT	6.45 ± 0.49	nd	4.07 ± 0.05	nd	550.01 ± 25.59
Thiourea	-	31.14 ± 5.29	-	-	-
EDTA	-	-	-	4.78 ± 0.28	-

^* ^IC_50_ value: concentration at which the DPPH and OH radicals were scavenged by 50%, absorbance was 0.5 for reducing power, iron(II) ions were chelated by 50%, respectively; ^** ^Results are calculated for sample concentrations of 10 µg/mL. Each value is expressed as mean ± SD. (n = 3); nd - not determined at tested concentrations; - not tested.

The influence of total polyphenol and individual polyphenolic subclass contents on antioxidant activities of tested *Micromeria* ethanolic extracts was also evaluated. The results showed very strong positive correlation between amounts of phenolic acids, tannins and total polyphenols and antioxidative activities determined in all assays, such as DPPH (*r* = 0.8492–0.9961) and OH scavenging activities (*r* = 0.9199–0.9998), reducing power (*r* = 0.9253–0.9860) and iron chelating activity (*r* = 0.7263–0.9936), as well as total antioxidant capacity (*r* = 0.7937–0.9847). Our results were consistent with the previous reports where significant contribution of the polyphenols to the antioxidant activity of medicinal plants was observed [37,38]. However, flavonoids were found in much lower percentages than other polyphenols and we could not show positive correlation between their amounts and antioxidant effects, therefore the obvious antioxidant capabilities of tested *Micromeria* species could not be related to the presence of flavonoids. 

## 3. Experimental

### 3.1. Plant material and extraction procedure

Aerial parts of investigated wild-growing *Micromeria* species were collected at the full flowering stage in June and July 2008 from three different locations in Croatia: *Micromeria croatica* (Pers.) Schott. (Veliko Libinje, 950 m a.s.l.), *Micromeria juliana* (L.) Bentham ex Reichenb. (Borovci, 50 m a.s.l) and *Micromeria thymifolia* (Scop.) Fritsch (Učka, 1,200 m a.s.l.). All plant samples were authenticated by the Department of Pharmacognosy, Faculty of Pharmacy and Biochemistry and Department of Botany and Botanical Garden, Faculty of Science (University of Zagreb, Croatia) where the voucher specimens have been deposited under the genus number 812a.

Air-dried and pulverized plant material (20.00 g) was extracted with 70% ethanol (200 mL) using an ultrasonic bath for 30 minutes. The extract was then filtered through Whatman No. 1 paper using a Büchner funnel and the residue was then re-extracted with the same solvent (200 mL) as described above. Obtained extracts were combined and then concentrated to dryness under vacuum at 50 °C using a rotary evaporator. The extraction yields for *M. croatica*, *M. juliana* and *M. thymifolia* were 21.60%, 19.25% and 18.28%, respectively.

### 3.2. Chemicals

Acetic acid, aluminium chloride, disodium hydrogen phosphate, ethanol, ethylenediaminetetraacetic acid (EDTA), hexamethylenetetramine, methanol, sodium carbonate, sodium citrate, sodium dihydrogen phosphate, sodium hydroxide, sodium nitrite, sodium phosphate, sulphuric acid, tannic acid (95%), thiourea were purchased from Kemika (Zagreb, Croatia). Ammonium molybdate, casein, 2-deoxy-D-ribose, 3-(2-pyridyl)-5,6-diphenyl-1,2,4-triazine-4′,4′′-disulfonic acid sodium salt (ferrozine), 2,2-diphenyl-1-picryl-hydrazyl (DPPH^·^), hydrogen peroxide, potassium ferricyanide, rosmarinic acid (96%), sodium acetate, and sodium molybdate were obtained from Sigma-Aldrich (St. Louis, MO, USA). Butylated hydroxytoluene (BHT, ≥99%), iron(II) chloride and quercetin-3-rutinoside (rutin, ≥95%) were obtained from Fluka (Buchs, Switzerland). Ascorbic acid (99%) and trichloroacetic acid (TCA) were purchased from Acros Organics (Geel, Belgium), Folin–Ciocalteu’s phenol reagent and 2-thiobarbituric acid (TBA) were obtained from Merck (Darmstadt, Germany). Iron(III) chloride and hydrochloric acid were obtained from Riedel-de Haën (Seelze, Germany) and POCh (Gliwice, Poland), respectively. All chemicals and reagents used were of analytical grade.

### 3.3. Phytochemical analyses of polyphenols

#### 3.3.1. TLC analysis

Thin-layer chromatographic (TLC) analysis of different polyphenol subclasses was performed on precoated silica gel 60 F_254 _TLC plate (Merck, Germany). Aliquots (10 μL) of 2% ethanolic extracts and 0.5% ethanolic solution of reference substances were manually applied on the plates which were then developed in vertical glass chambers previously saturated with the mobile phases: ethyl acetate-formic acid-acetic acid-water (100:11:11:27, v/v/v/v) [39], diisopropyl ether–acetone–water–formic acid (50:30:10:10, v/v/v/v) [40] and ethyl formate-formic acid-water (80:10:10, v/v/v) [39] for analysis of flavonoids, phenolic acids and tannins, respectively. After development plates were dried in a stream of air for a few minutes. Flavonoids and phenolic acids were detected under UV light at 365 nm after spraying them with natural products-polyethylene glycol reagent (1% methanolic solution of 2-aminoethyl diphenylborinate and 5% ethanolic solution of PEG 4000, NST/PEG). For visualisation of tannins in visible light, plates were sprayed with 10% ethanolic iron(III) chloride.

#### 3.3.2. Determination of total phenolic acids

Determination of hydroxycinnamic acid derivatives was performed according to procedure described in European Pharmacopoeia [41]. Briefly, the powdered plant material (0.20 g) was extracted with 50% ethanol (80 mL) under a reflux condenser in a boiling water bath for 30 min. The cooled extract was filtered, the filter rinsed with ethanol, and then the combined filtrate and rinsing solution was diluted to 100.0 mL with 50% ethanol. An aliquot of the extract (1.0 mL) was mixed with 0.5 M hydrochloric acid (2 mL), Arnow reagent (10% aqueous solution of sodium nitrite and sodium molybdate, 2 mL), and 8.5% sodium hydroxide (2 mL) and diluted to 10.0 mL with water. The absorbance of the test solution was measured immediately at 505 nm against sample blank. The percent of total hydroxycinnamic acid content was calculated and expressed as rosmarinic acid, according to the following expression: *(%) = A × 2.5/m,* where *A* is the absorbance of the test solution at 505 nm and *m* is the mass of the sample, in grams. Analysis of each sample was performed in triplicate.

#### 3.3.3. Determination of total flavonoids

The total flavonoid contents of three *Micromeria* species were determined using the spectrophotometric method of Christ and Müller [42]. Each powdered plant sample (0.20 g) was mixed with acetone (20 mL), 25% hydrochloric acid (2 mL) and 0.5% hexamethylenetetramine solution (1 mL) and heated under reflux in a water bath for 30 min. The extract was filtered and re-extracted twice with 20 mL of acetone for 10 min. Filtrates were combined and made up to 100.0 mL with acetone. An aliquot of the acetone extract (20 mL) was mixed with water (20 mL) and then extracted with three portions of ethyl acetate (each of 15 mL). The combined ethyl acetate layers were washed twice with water then filtered and diluted to 50.0 mL. To this solution (10.0 mL) 0.5% solution of sodium citrate (0.5 mL) and 2% aluminium chloride solution (in 5% methanolic solution of acetic acid, 2 mL) was added and then diluted to 25.0 mL with 5% methanolic solution of acetic acid. The mixture was allowed to stand for 45 min and the absorbance was measured at 425 nm. A sample solution prepared in the same manner but without addition of aluminium chloride solution served as a blank. All determinations were performed in triplicate. The percentage content of flavonoids, expressed as quercetin, was calculated from the equation: *(%) = A × 0.772/m*, where *A* is the absorbance of the test solution at 425 nm and *m* is the mass of the sample, in grams.

#### 3.3.4. Determination of total polyphenols and tannins

Total polyphenolic compounds were assayed using the Folin-Ciocalteu’s reagent and tannin content was quantified by the casein precipitation method, as described by Schneider [43]. For this purpose, the powdered plant sample (0.25 g) was extracted with 30% methanol (80 mL) in a water bath (70 °C) for 15 min. After cooling and filtration, the extract was made up to 100.0 mL with 30% methanol. Solution 1 was prepared by mixing plant extract (2.0 mL) with distilled water (8 mL) and sodium acetate buffer (pH = 5, 10 mL). Solution 2 was obtained by shaking solution 1 (10.0 mL) with casein (50 mg) for one hour, followed by filtration. To each solution (solution 1 and solution 2, respectively, 1.0 mL), Folin-Ciocalteu’s phenol reagent (0.5 mL) was added, and the resulting mixtures were made up to 10.0 mL with saturated 33% sodium carbonate solution. The absorbance was read at 720 nm using water as a blank. Absorbance value obtained for solution 1 corresponds to total polyphenol content, while the difference between the absorbances in the total polyphenol analysis (solution 1) and polyphenols unadsorbed on casein (solution 2) corresponds to amount of tannins in plant samples. Quantification was done with respect to the standard calibration curve of tannic acid (concentration range 10–60 μg/mL) and the results were expressed in percentage of weight of the dry sample. All assays were carried out in triplicate.

### 3.4. Evaluation of antioxidant activity

#### 3.4.1. 2, 2-Diphenyl-1-picrylhydrazyl radical (DPPH•) radical scavenging assay

The free radical scavenging activities of the samples were measured using the stable DPPH radical, according to the method of Blois [44]. Briefly, 0.1 mM solution of DPPH^•^ in ethanol was prepared and this solution (1 mL) was added to sample solution in ethanol (3 mL) at different concentrations (0.63–80 µg/mL). The mixture was shaken vigorously and left to stand for 30 min in the dark, and the absorbance was then measured at 517 nm. The capability to scavenge the DPPH^•^ radical was calculated using the following equation: *(%) = [(A_0_ − A_1_)/A_0_] × 100*, where *A_0_* is the absorbance of the control reaction and *A_1_* is the absorbance in the presence of sample, corrected for the absorbance of sample itself. Butylated hydroxytoluene (BHT) was used for comparison. All determinations were done in triplicate.

#### 3.4.2. Hydroxyl radical (OH^•^) scavenging assay

Hydroxyl radicals were generated by a Fenton reaction (Fe^3+^-ascorbate-EDTA-H_2_O_2 _system), and the scavenging capacity towards the hydroxyl radicals was measured by using a deoxyribose method as described by Halliwell *et al.* [45] with a slight modification. The reaction mixture contained, in a final volume of 1 mL, 2-deoxy-2-ribose (2.8 mM), phosphate buffer (0.1 mM, pH 7.4), iron(III) chloride (20 μM), EDTA (100 μM), hydrogen peroxide (500 μM), ascorbic acid (100 μM) and various concentrations (10–640 μg/mL) of the test sample or the reference compound. After incubation for 1 h at 37 °C, an aliquot of the reaction mixture (0.5 mL) was added to 2.8% TCA solution (1 mL), followed by TBA solution (1% in 50 mM sodium hydroxide, 1 mL) and then the mixture was heated (20 min at 90 °C) to develop the colour. After cooling, the absorbance was measured at 532 nm against an appropriate blank solution. All experiments were performed in triplicate. Hydroxyl radical scavenging activity was expressed as the percentage of inhibition of 2-deoxyribose oxidation by hydroxyl radicals, according to the following equation: *(%) = [A_0_ − (A_1_ − A_2_)]/A_0_ × 100*, where: *A_0_* is the absorbance of the control without a sample, *A_1_* is the absorbance in the presence of the sample and deoxyribose and *A_2_* is the absorbance of the sample without deoxyribose. Thiourea was used as a positive control. 

#### 3.4.3. Reducing power assay

The reducing power of samples was determined by the method of Oyaizu [46]. An aliquot of the sample (1.0 mL) at various concentrations (1.25–80 μg/mL) was mixed with phosphate buffer (0.2 M, pH 6.6, 2.5 mL) and 1% potassium ferricyanide (2.5 mL). The mixture was incubated at 50 °C for 20 min. After adding 10% trichloroacetic acid (2.5 mL), the mixture was centrifuged at 650 rpm for 10 min. The supernatant (2.5 mL) was mixed with distilled water (2.5 mL) and 0.1% iron(III) chloride (0.5 mL), and the absorbance was measured at 700 nm using an appropriate blank. Assays were carried out in triplicate. BHT was used as a reference.

#### 3.4.4. Metal ion chelating assay

The ability of samples to chelate iron(II) ions was estimated using the method reported by Gülçin [47] and compared with that of the reference chelator agent EDTA. Different concentrations of the sample (final concentration 20–640 μg/mL) were added to a solution of 2 mM iron(II) chloride (0.05 mL). The reaction was initiated by the addition of 5 mM ferrozine (0.2 mL) and the volume of the mixture was finally adjusted to 4 mL with ethanol, shaken vigorously and left standing at room temperature for 10 min. After the mixture had reached equilibrium, the absorbance of the solution was measured spectrophotometrically at 562 nm. The percentage of inhibition of ferrozine–Fe^2+^ complex formation was calculated using the formula given below: *(%) = [A_0_ − (A_1_ − A_2_)]/A_0_ × 100*, where *A_0_* is the absorbance of the control, containing iron(II) chloride and ferrozine only, *A_1_* is the absorbance in the presence of the tested sample and *A_2_* is the absorbance of the sample under identical conditions as *A_1_* with water instead of iron(II) chloride solution. All assays were done in triplicate.

#### 3.4.5. Total antioxidant capacity assay

The total antioxidant capacities of three *Micromeria* extracts and their active constituents were evaluated by the phosphomolybdenum method, according to the procedure of Prieto *et al.* [36]. An aliquot of the sample solution in ethanol (0.4 mL) was combined in a vial with reagent solution (0.6 M sulfuric acid, 28 mM sodium phosphate and 4 mM ammonium molybdate, 4 mL). The effective concentrations of the sample in the reaction mixtures were in the range of 10–80 μg/mL. The vials were capped and incubated in a water bath at 95 °C for 90 min. After cooling the mixture to room temperature, the absorbance was measured at 695 nm against a blank. All assays were run in triplicate. The antioxidant capacity of the sample was expressed as equivalents of ascorbic acid (AAE), utilizing a calibration curve of ascorbic acid in the concentration range from 1.25 μg/mL to 160 μg/mL. 

### 3.5. Statistical analysis

All experiments were carried out in triplicate, and the results are expressed as mean ± standard deviation (SD). Differences were estimated by Student's t-test and the values p < 0.05 were considered statistically significant. The concentrations of samples that provide 50% inhibition (IC_50_) were obtained by interpolation from linear regression analysis. Correlation analyses between the various antioxidant assays and phenolic contents were performed calculating the Pearson’s correlation coefficient (*r*). All statistical analyses were carried out using Microsoft Excel 2000 software. 

## 4. Conclusions

In this work, the detailed polyphenolic contents of *Micromeria croatica*, *M. juliana* and *M. thymifolia* and their related antioxidant activities were demonstrated for the first time. Our results provide evidence that all the tested plant species are capable of directly quenching free radicals to terminate the radical chain reaction, acting as reducing agents, and chelating transition metals to suppress the initiation of radical formation. Considerable antioxidant abilities of *Micromeria* ethanolic extracts are mainly attributed to the high level of polyphenolic substances. Rosmarinic acid, as the most abundant polyphenolic constituent found, is very likely to contribute largely to the observed effects. Among tested plants, *M. croatica* is considered as the richest source of antioxidant polyphenols and to be the most potent one. Consequently, *Micromeria* species may have a great relevance in the prevention and therapies of diseases in which free radicals and oxidants are implicated. Besides the health aspect, these plants can also be good candidates for the development of antioxidant food additives. The results presented in this paper have inspired our current study for isolation and structure elucidation of their active components.
